# Effect of hyperglycemia and rosiglitazone on renal and urinary neprilysin in *db*/*db* diabetic mice

**DOI:** 10.14814/phy2.14364

**Published:** 2020-02-05

**Authors:** Laale F. Alawi, Sana E. Emberesh, Brenda A. Owuor, Harshita Chodavarapu, Rucha Fadnavis, Salim S. El‐Amouri, Khalid M. Elased

**Affiliations:** ^1^ Department of Pharmacology and Toxicology Boonshoft School of Medicine Wright State University Dayton OH USA; ^2^ Boonshoft School of Medicine Department of Neuroscience Cell Biology and Physiology Wright State University Dayton OH USA

**Keywords:** biomarker, CKD, diabetes, rosiglitazone

## Abstract

Alteration in renin‐angiotensin system (RAS) has been implicated in the pathophysiology of diabetic kidney disease (DKD). The deleterious actions of angiotensin II (Ang II) could be antagonized by the formation of Ang‐(1–7), generated by the actions of angiotensin‐converting enzyme 2 (ACE2) and neprilysin (NEP). NEP degrades several peptides, including natriuretic peptides, bradykinin, amyloid beta, and Ang I. Although combination of Ang II receptor and NEP inhibitor treatment benefits patients with heart failure, the role of NEP in renal pathophysiology is a matter of active research. NEP pathway is a potent enzyme in Ang I to Ang‐(1–7) conversion in the kidney of ACE2‐deficient mice, suggesting a renoprotective role of NEP. The aim of the study is to test the hypothesis that chronic hyperglycemia downregulates renal NEP protein expression and activity in *db*/*db* diabetic mice and treatment with rosiglitazone normalizes hyperglycemia, renal NEP expression, and attenuates albuminuria. Mice received rosiglitazone (20 mg kg^−1^ day^−1^) for 10 weeks. Western blot analysis, immunohistochemistry, and enzyme activity revealed a significant decrease in renal and urinary NEP expression and activity in 16‐wk *db*/*db* mice compared with lean control (*p* < .0001). Rosiglitazone also attenuated albuminuria and increased renal and urinary NEP expressions (*p* < .0001). In conclusion, data support the hypothesis that diabetes decreases intrarenal NEP, which could have a pivotal role in the pathogenesis of DKD. Urinary NEP may be used as an index of intrarenal NEP status. The renoprotective effects of rosiglitazone could be mediated by upregulation of renal NEP expression and activity in *db*/*db* diabetic mice.

## Graphical abstract

Decreased intrarenal NEP, which could have a pivotal role in the pathogenesis of diabetic kidney disease. Urinary NEP could be an index of intra‐renal NEP status. The renoprotective effects of rosiglitazone could be mediated by the up‐regulation of renal NEP expression and activity in db/db diabetic mice.

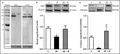

## 1 INTRODUCTION

Diabetic kidney disease (DKD) is one of the most frequent complications of both types of diabetes and the leading cause of end‐stage renal disease (ESRD) in the Western world (Tuttle et al., 2014). DKD is characterized by a progressive decline in the glomerular filtration rate, which is often accompanied by albuminuria and could ultimately lead to ESRD (Alicic, Rooney, & Tuttle, 2017). DKD also increases the risk of cardiovascular disease (Fox et al., 2012), which is considered to be one of the main cause of morbidity and mortality in diabetic patients (Einarson, Acs, Ludwig, & Panton, 2018; Saran et al., 2016). Risk factors for chronic kidney disease (CKD) include hypertension, diabetes, proteinuria, age, and obesity. Due to the increased incidence and prevalence of type 2 diabetes worldwide, diabetic patients with end‐stage renal disease have increased in the recent decades, creating a need for the development of novel treatment and early biomarkers for detection of DKD. The pathogenesis of DKD is complex and has not yet been well elucidated. Glomerular hyperfiltration is commonly observed in early stages of DKD, which raises the glomerular capillary pressure as a result of hemodynamic functional abnormalities (Brewster, Perazella, & Setaro, 2003; Campbell & Yacoub, 2015). Renin angiotensin system (RAS) plays a central role in the control of blood pressure, and medications targeting the RAS are the most validated clinical strategies for slowing CKD progression (Campbell & Yacoub, 2015; Márquez, Riera, Pascual, & Soler, 2015). This dysregulation in the RAS system leads to increased Ang II expression along with an increase in sensitivity to its effects in the kidney. An Ang‐II‐dependent upregulation of atrial natriuretic peptide (ANP) is also found to develop in DKD (Campbell & Yacoub, 2015; Ortola, Ballermann, Anderson, Mendez, & Brenner, 1987). Ang II plays a central role in causing renal damage (Márquez et al., 2015). A variety of mechanisms have been suggested by which Ang II can induce renal damage, however, the deleterious actions of Ang II can be antagonized by the formation of a vasodilator, Angiotensin (1–7), which is generated mainly by the actions of ACE2 and NEP (Donoghue et al., 2000; Rice, Thomas, Grant, Turner, & Hooper, 2004).

ACE2 has been found to be downregulated in kidneys of animal models of diabetes (Yamaleyeva et al., 2012; Ye et al., 2006). The importance of ACE2 as a renoprotective enzyme is well established by studies showing exacerbation in kidney injury following pharmacologic or transgenic deficiency of ACE2 (Soler et al., 2007; Ye et al., 2006). Several studies have shown the protective roles for ACE2 against cardiovascular and renal dysfunction in animal model of diabetes, hypertension, and heart failure (Batlle, Wysocki, Soler, & Ranganath, 2012).

Neutral endopeptidase (NEP), also known as CD10, enkephalinase, and common acute lymphoblastic leukemia antigen (CALLA), has a molecular weight of approximately 90–110 kDa with a short end N‐terminal cytoplasmic domain and a large C‐terminal extracellular domain. NEP was initially discovered in the brush border membranes of a rabbit kidney as a metalloenzyme that degrades the insulin beta chain (Kerr & Kenny, 1974). Subsequent research has found NEP to be widely expressed in many organs, including adrenal glands, intestines, lungs, brain, and proximal renal tubules (Judge, Haynes, Landray, & Baigent, 2015; Li, Booze, & Hersh, 1995). Apart from Ang I, NEP is also involved in the degradation of several biologically active peptides, such as bradykinin, atrial natriuretic peptide (ANP), C‐type natriuretic peptide (CNP), and B‐type natriuretic peptide (BNP) (Judge et al., 2015; Watanabe, Nakajima, Shimamori, & Fujimoto, 1997). Recent research has found that an active soluble neprilysin (sNEP) level is elevated in the plasma of chronic and acute heart failure patients (Bayés‐Genís et al., 2015; Bayes‐Genis, Prickett, Richards, Barallat, & Lupón, 2016). It has been hypothesized that an elevated level of NEP can be used as a biomarker to assess the prognosis and condition of acute and chronic heart failure patients (Bayes‐Genis et al., 2015; Bayés‐Genís et al., 2015; Núñez et al., 2017). Similarly, changes in NEP expression and activity have also been suggested in other diseases. For example, a recent study conducted using a monkey model of type‐1 diabetes has found that NEP expression is decreased in the hippocampus with a concurrent increase in Aβ peptide deposits (Morales‐Corraliza et al., 2016). NEP has also been involved and proposed as a prognostic biomarker of various malignancies, such as renal neoplasms, malignant melanoma, and breast carcinoma (Maguer‐Satta, Besançon, & Bachelard‐Cascales, 2011). Therefore, controlling ACE2 and NEP expression could be beneficial not only for predicating their associated diseases condition but also as a treatment strategy.

Thiazolidinediones (TZDs), such as rosiglitazone and pioglitazone, are high affinity agonists of the peroxisome proliferator‐activated receptor gamma (PPAR‐γ). They are used as insulin sensitizers to control hyperglycemia in type 2 diabetes (Tontonoz & Spiegelman, 2008) and to protect kidneys from diabetic and nondiabetic injuries by reducing microalbuminuria (Bakris et al., 2006; Sarafidis, Stafylas, Georgianos, Saratzis, & Lasaridis, 2010; Sugawara et al., 2012). Rosiglitazone has also been found to have a cardioprotective effect upon the cardiac muscle in the diabetic rat model (Abou Daya, Abu Daya, Nasser Eddine, Nahhas, & Nuwayri‐Salti, 2015; Yue et al., 2005). However, the exact mechanism of how rosiglitazone act as a cardio‐ or renoprotective is still unknown. Our previous study showed that rosiglitazone treatment of type 2 diabetic *db*/*db* mice increased renal ACE2 expression levels while attenuating urinary albumin and ACE2 excretion (Chodavarapu et al., 2013). This suggests that the renoprotective effect of rosiglitazone could be mediated by the increase in RAS enzyme ACE2. However, whether other RAS enzymes, such as NEP can provide a renoprotection against diabetic nephropathy is unknown. It is important to mention that both NEP and ACE2 can counteract the effects of ACE and Ang II via the formation of Ang‐(1–7). Our recent studies demonstrated increased urinary NEP in patients with type 2 diabetes compared with nondiabetic volunteers (Gutta et al., 2018). This cohort of diabetic patients were treated with different classes of antidiabetic medications including PPAR‐γ agonists. Therefore, the aim of this study was to address two questions: (a) are renal NEP protein expression and activity altered in *db*/*db* diabetic mice and (b) does normalizing glycemia with rosiglitazone treatment modulate renal NEP protein expression and activity? This approach will directly dissect the effect of hyperglycemia and PPAR‐γ agonist on renal and urinary NEP expression and activity.

## 2 MATERIALS AND METHODS

### 2.1 Reagents

Rosiglitazone was purchased from LKT Laboratories, Inc. (St. Paul, MN, USA). Primary polyclonal goat anti‐NEP (Cat # AF1126) and donkey anti‐goat secondary antibody (Cat # HAF017) were purchased from R&D Systems. CY^3^ conjugated donkey anti‐goat secondary antibody (code # 705‐165‐147), CY^3^ conjugated donkey anti‐rabbit secondary antibody (code # 711‐165‐152) and FITC conjugated donkey anti‐goat secondary antibody (code # 705‐095‐147) were purchased from Jackson Immunoresearch. Primary polyclonal rabbit anti‐ACE2 form Sigma (Cat # HPA000288). Mouse Albumin ELISA kit was from Bethyl Laboratories (Cat # E90‐134). Mouse Neprilysin DuoSet ELISA development kit from R&D systems (Cat # DY1126). SensoLyte^®^ 520 NEP activity assay kit from AnaSpec EGT group (Cat# 72223).

### 2.2 Animals

All animal studies were performed under a protocol approved by the Institutional Animal Care and Use Committee at Wright State University. Six‐week‐old male *db/db* diabetic mice (C57BL/KsJ (BKS.Cg‐Dock7*m* +/+ Lepr*^db^*/J) and their age‐matched nondiabetic lean control mice (*db/m*) were obtained from Jackson Laboratories (Bar Harbor, ME, USA). Mice were housed individually in cages with ad libitum access to food and water and maintained at room temperature (22°C) with 12:12 hr light‐dark cycles. For tissue acquisition for histology, mice were deeply anesthetized with sodium pentobarbital (60 mg/kg, administered intraperitoneally) and then perfused transcardially with cold PBS and 4% paraformaldehyde. Every effort was made to minimize suffering and pain. For tissue acquisition for western blot, ELISA and enzyme activity, mice were euthanized by decapitation and kidneys were dissected and stored at –80°C.

Kidney tissues from NEP gene knockout mice (NEP^−/−^) and their littermate wild‐type (NEP^+/+^) were obtained from Dr. Bao Lu (Harvard Medical School). The kidney tissues were homogenized on ice in phosphate‐buffered saline (PBS) containing protease inhibitor (Complete lysis M, Roche diagnostics). Homogenates were then centrifuged at 10,000*g* for 10 min at 4°C to remove cellular debris. The supernatants were collected, aliquoted, and stored at (−80°C).

### 2.3 Treatment with rosiglitazone

Rosiglitazone was purchased from LKT Laboratories, Inc. and used to enrich the diet by Harlan Teklad. Seven‐week‐old mice were randomly assigned to three different groups: (a) Nondiabetic lean control mice fed normal chow; (b) Nondiabetic control mice fed rosiglitazone diet (20 mg kg^−1^ day^−1^); (c) *db*/*db* diabetic mice fed normal chow; and (d) *db*/*db* diabetic mice fed rosiglitazone diet (20 mg kg^−1^ day^−1^) for 10 weeks. Body weight, food intake, water intake, urine output, and blood glucose were monitored weekly. After the treatment period, mice were euthanized by decapitation. Trunk blood samples were collected into prechilled heparinized tubes, centrifuged at 10,000*g*, at 4°C for 10 min. Plasma was collected, aliquoted, and stored at (−80°C) for later use. Kidneys were removed, frozen in liquid nitrogen, and stored at (−80°C).

### 2.4 Measurement of blood glucose levels

FreeStyle Blood Glucose Test Strips and Monitoring System were used to determine blood glucose levels weekly. A cut was made at the tip of the tail to draw blood samples. Values were expressed in mg/dl.

### 2.5 Urine samples collection

Mice were placed individually in separate Tecniplast metabolic cages and 24 hr urine samples were collected at baseline and weekly after treatment through the study. To avoid protein degradation, urine samples were collected in the presence of 20 μl protease inhibitor containing PMSF (Roche Diagnostics). Urine samples were centrifuged at 1,000*g* for 5 min at 4°C to remove cellular debris. The supernatants were aliquoted and stored at −80°C for later analysis.

### 2.6 Urinary albumin assay

Urinary albumin was measured using a mouse ELISA kit purchased from Bethyl Laboratories as described previously (Chodavarapu et al., 2013; Somineni, Boivin, & Elased, 2014). The standard dilutions were prepared according to the kit's protocol and diluted with sample/conjugate buffer. Urine samples were diluted with sample/conjugate buffer in the ratio 1:500. The assay was performed as per the instructions provided in the kit. Final absorbance was read at 450 nm in a Fusion Packard plate reader (Packard BioScience). Unknown urinary albumin concentrations were determined from a standard curve plotted using assay standards in the range 7.8–500 ng/ml.

### 2.7 Renal and urinary neprilysin (NEP) measurements

The Mouse neprilysin Duoset kit purchased from R&D systems (Minneapolis, MN, USA) was used for quantitative measurement of NEP levels in 24 hr urine, kidney lysate, and plasma samples. The plate was coated using diluted capture antibody and incubated overnight at room temperature (RT). The plate was then washed and blocked for an hour at RT using reagent diluents buffer. Standard dilutions were prepared according to the kit's protocol, and samples were diluted with sample/conjugate buffer as follows: urine samples in the ratio 1:50, kidney lysate samples in the ratio 1:100, and plasma samples in the ratio 1:4, then added to the wells and incubated for 2 hr at RT. Steps for detection antibody, adding working solutions of Streptavidin‐HRP, and substrate solution (TMB) were performed as stated in the kit's protocol. Finally, the reaction was stopped by H_2_SO_4_ and absorbance was read at 450 nm in a Fusion Packard plate reader (Packard BioScience). Unknown NEP concentrations were determined from a standard curve plotted using assay standards in the range 0.093–6 ng/ml.

### 2.8 Measurement of NEP activity

The SensoLyte 520 NEP activity assay kit purchased from ANASPEC was used to measure NEP activity in kidney lysate samples with some modifications. The principle of the assay is based on the cleavage of NEP of the quenched 5‐FAM/QXL^™^ FRET substrate into two separate fragments to produce 5‐FAM fluorescence. Kidney lysates from NEP knockout (NEP KO) mice and the specific NEP inhibitor, thiorphan were used to determine the specificity of NEP activity assay kit. For each reaction, the kidney lysates (30–35 µg protein) were incubated with and without the NEP inhibitor, thiorphan (10 mM), in black‐colored 96‐well plate (Corning Inc), followed by 50 µl of the reaction substrate (5‐FAM/QXLTM‐520 neprilysin substrate, 1 mM). The fluorescence was measured immediately and every minute over 10 min using Synergy H1 microplate reader (BioTek) at excitation/emission = 490/520 nm wavelength.

### 2.9 Western blot analysis

After the treatment period, mice were euthanized by decapitation and whole kidneys were quickly dissected and homogenized in EDTA‐free lysis buffer containing protease inhibitor cocktail (Roche Diagnostics) and freshly added 2.5 mM PMSF (Millipore Sigma). Kidney lysates were centrifuged at 10,000*g* for 10 min at 4°C. BioRad reagent (BioRad) was used to measure the total protein concentration of each sample. Total protein concentration was determined in the supernatant of each samples using BSA as a standard and BioRad reagent (BioRad). Samples were solubilized in Laemmle's buffer with β‐mercaptoethanol. Thirty micrograms (30 µg) of protein from kidney lysate samples, and three micrograms (3 µg) of creatinine from urine samples were loaded in each well and resolved by electrophoresis on a 10% SDS‐PAGE gel for an hour. Separated proteins were transferred (Bio‐Rad transfer apparatus) to a 0.2 μm PVDF membrane. The membranes were blocked for an hour using nonfat dry milk in TBS‐T buffer, and then incubated with NEP primary antibody (1:500) goat anti‐NEP (1:1,000, Cat # AF1126, R&D Systems) over night at 4°C. Membranes were then washed and incubated with donkey anti‐goat secondary antibody (1:2000). Detection of protein bands was accomplished using SuperSignal chemiluminescent substrate (Thermo Scientific) and visualized using a Fujifilm image analyzer (LAS 3000, image Quant). The relative amounts of proteins of interest were determined by normalizing to β‐actin in kidney lysate samples, and by creatinine values in 24 hr‐urine samples. Western blot images were quantified using Image lab 4.0 software using β‐actin as an internal loading control.

### 2.10 Kidney histology and immunohistochemistry

Paraffin‐embedded kidney sections (4 m) were deparaffinized in xylene and rehydrated in graded alcohol concentrations. Sections were subsequently boiled with 10 mM sodium citrate in a water bath for 30 min. The sections were then blocked in 3% normal donkey serum (diluted in PBS containing 0.1% Triton‐X) for 1 hr at 4°C, and then incubated overnight at 4°C with diluted primary antibodies as follows: goat anti‐NEP (1:500, Cat # AF1126, R&D Systems) and rabbit anti‐ACE2 (1:500, Cat # HPA000288, Sigma). Then, the sections were incubated with CY^3^ conjugated donkey anti‐goat secondary antibody (1:500, code # 705‐165‐003, Jackson Immunoresearch). For the co‐localization, FITC conjugated donkey anti‐goat secondary antibody (1:500, code # 705‐095‐147, Jackson Immunoresearch) and CY^3^ conjugated donkey anti‐rabbit secondary antibody (1:500, code # 711‐165‐152, Jackson Immunoresearch) were used and incubated for 2 hr at 4°C. Sections were mounted using a vectashield‐mounting medium (Vector). Images were acquired using a fluorescence microscope (Optronics).

For morphological evaluation of kidney injury, sections were stained with periodic acid‐Schiff (PAS) by AML laboratories. Sections were examined under bright color field and images were acquired using a 40× objective on the Cytation 5 Automated Microscope (BioTek Instruments Inc.).

### 2.11 Statistical analysis

Statistical analysis was performed using GraphPad Prism software 5.01. The data were assessed by repeated measures two‐way ANOVA followed by Bonferroni's multiple comparison tests. One‐way ANOVA was used for more than two groups. Unpaired student's *t*‐*t*est was used to evaluate the differences between two groups. A value of *p* < .05 was considered statistically significant. All the results are presented as mean ± *SEM*.

## 3 RESULTS

### 3.1 Effects of rosiglitazone on blood glucose

Six‐week‐old *db/db* mice had significantly high blood glucose levels (575.6 ± 20.2 mg/dl) compared to age‐matched normal non‐diabetic control mice (135.3 ± 3.8 mg/dl) (*p* < .0001). As expected, the blood glucose levels in the *db*/*db* mice were consistently increased over the study period; and as shown in Table 1, the blood glucose concentrations of 16 week old *db*/*db* mice were significantly higher compared with the age‐matched lean controls (Chodavarapu, Kablan, Salem, & Elased, 2011). During the 10‐week treatment period, rosiglitazone significantly reduced blood glucose level and after 1 week it retrieved blood glucose to the normal level (113.8 ± 6.5 mg/dl) (Table 1). However, rosiglitazone had no effect on blood glucose in normal nondiabetic control mice as shown previously (113.9 ± 2.6 mg/dl) (Chodavarapu et al., 2013).

**Table 1:** General parameters of Lean, Lean + Rosiglitazone, *db/db*, and *db/db*+ Rosiglitazone mice

Mice strain	Lean	Lean + Rosiglitazone	*db/db*	*db/db*+ Rosiglitazone
Age (Weeks)	16	16	16	16
Duration of treatment (weeks)	10	10	10	10
Group size (*n*)	6	6	6	6
Body weight (g)	27.8 ± 1.6	30.3 ± 0.7	39.7 ± 0.7*$	64.9 ± 0.7*, $
Blood glucose (mg/dl)	135.3 ± 3.8	113.9 ± 2.6	575.6 ± 20.2*$	113.8 ± 6.5#
Food intake (g/day)	3.4 ± 0.1$	4.6 ± 0.2	6.8 ± 0.8*$	5.9 ± 0.2$
Water intake (ml/day)	5.9 ± 0.3$	7.3 ± 0.6	31.4 ± 3.7*$	8.4 ± 0.3#
Urine output (ml/day)	0.9 ± 0.2	0.86 ± 0.6	25.4 ± 1.8*$	1.2 ± 0.2#

Note

Values represent mean ± *SEM*.

* *p* < .05 versus age‐matched lean mice.

^$^ *p* < .05 versus age‐matched lean + rosiglitazone mice.

^#^ *p* < .05 versus Age‐matched *db/db* mice were considered statistically significant.

### 3.2 Effects of rosiglitazone on urinary albumin excretion and kidney injury

**Figure 1:**
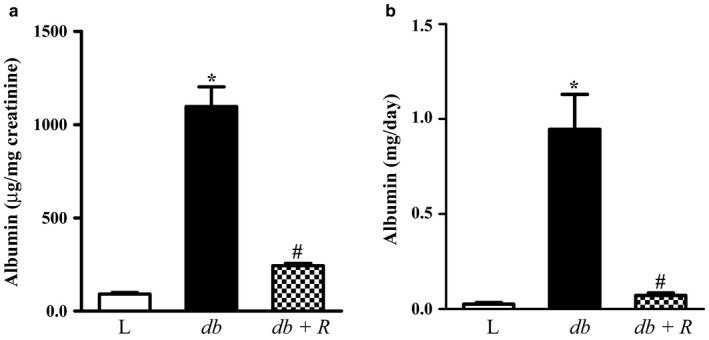
Chronic treatment with rosiglitazone for 10 weeks reduced the increased urinary albumin excretion in 16‐week‐old *db/db* diabetic mice. Albuminuria in lean control (L), *db*/*db* diabetic (*db*), and *db*/*db* diabetic mice treated with rosiglitazone (*db+ *R) was expressed as albumin/creatinine ratios (a) or as 24‐hr albuminuria (b). Data are expressed as means ± *SEM* (*n* = 6, *p < .0001 vs. lean controls, ^#^ *p* < .0001 vs. untreated *db*/*db* diabetic mice)

The urine albumin test or albumin/creatinine ratio (ACR) is an indicator used to screen for damage to the kidney structures due to conditions such as diabetes and high blood pressure. There was a significant increase in urinary albumin/creatinine ratio in *db/db* diabetic mice compared with aged‐matched lean normal nondiabetic mice (Figure 1a, *p* < .0001). Twenty‐four‐hour albumin excretion was significantly higher in *db*/*db* mice compared to lean control mice (Figure 1b, *p* < .0001). After 10 weeks of treatment with rosiglitazone, urinary albumin excretion in *db/db* mice significantly decreased compared to the untreated *db*/*db* diabetic mice (Figure 1a and b, *p* < .0001).

**Figure 2:**
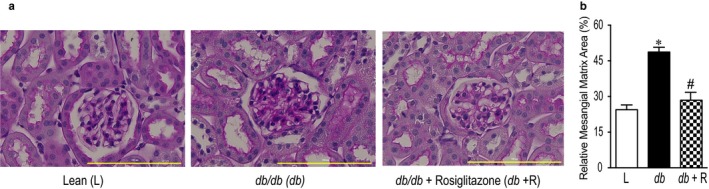
(a) Representative light microscopy images of kidney section from lean control (L), *db*/*db* diabetic (*db*), and *db*/*db* diabetic mice treated with rosiglitazone (*db+ *R) stained with periodic acid‐ Schiff (PAS). Magnification: 40×, scale bars:100 μm. (b) Relative mesangial matrix area (%) significantly increased in *db*/*db* mice and was attenuated by treatment with rosiglitazone for 10 weeks. Each bar represents mean ± *SEM* (*n* = 5–6, *p < .0001 vs. lean controls; ^#^ *p* < .001 vs. untreated *db/db* diabetic mice)

Renal mesangial expansion is one of the histological characteristics of diabetic nephropathy. The mesangial matrix was identified by the presence of PAS‐positive and nuclei‐free areas in the mesangium. Glomerular tufts of *db*/*db* mice revealed a significant increase in mesangial expansion, which was significantly reduced after treatment with rosiglitazone (Figure 2, *p* < .001).

### 3.3 Effects of rosiglitazone on renal and urinary NEP expression

**Figure 3:**
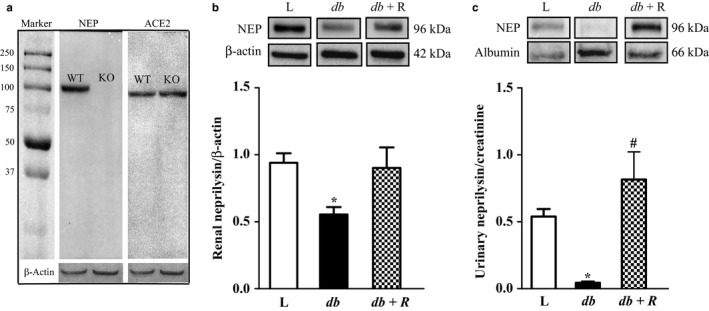
Renal and urinary NEP protein expressions were decreased in 16‐week‐old *db/db* diabetic mice. To validate the specificity of NEP antibody, western blot analysis of NEP and ACE2 were performed in kidney lysates obtained from NEP KO & NEP WT mice (a). Western blot analysis of (a) renal NEP and β‐actin; (c) urinary NEP and albumin in lean control (L), *db*/*db* diabetic (*db*) and *db*/*db* diabetic mice treated with rosiglitazone for 10 weeks (*db+ R*). One‐way ANOVA using Bonferroni's Multiple Comparison Test showed a significant decrease in the *db*/*db* diabetic mice compared to lean controls. Rosiglitazone normalized the decreased renal NEP and increased urinary NEP excretion. Values are represented as mean ± *SEM* (*n* = 6–11, *p < .001 vs. lean controls; ^#^ *p* < .0001 vs. untreated *db/db* diabetic mice)

In order to characterize the specificity of the NEP antibody used in this study, we carried western blotting with kidney tissues from wild‐type mice (WT) and genetically modified mice lacking NEP gene (NEP KO). As shown in Figure 3a, the NEP antibody generated a distinct single band around 96 kDa in NEP wild‐type kidney lysate, which is the expected size, while this band was absent in NEP KO mice. In addition, ACE2 bands appeared in both WT and NEP KO mice. This indicates that the NEP antibody used in this study is specific and selective.

We then evaluated NEP protein expression in urine and renal tissues of lean control, untreated *db*/*db* diabetic mice, and rosiglitazone treated *db/db* diabetic mice. Immunoreactive bands for renal NEP protein expression were detected in all groups at ~96 kDa. Renal NEP was significantly decreased in untreated *db*/*db* diabetic mice compared to lean control mice (Figure 3b, *p* < .001). Treatment with rosiglitazone normalized hyperglycemia and significantly increased renal NEP protein expression in the treated *db/db* compared to the untreated *db*/*db* mice (Figure 2b, *p* < .001). Similarly, urinary NEP protein expression was significantly decreased in untreated *db*/*db* diabetic mice compared to the lean nondiabetic control mice (Figure 3c, *p* < .001). Moreover, treatment with rosiglitazone significantly increased urinary NEP protein expression in the diabetic group compared with untreated *db/db* diabetic mice (Figure 3c, *p* < .001).

### 3.4 Effect of rosiglitazone on renal and urinary NEP content

**Figure 4:**
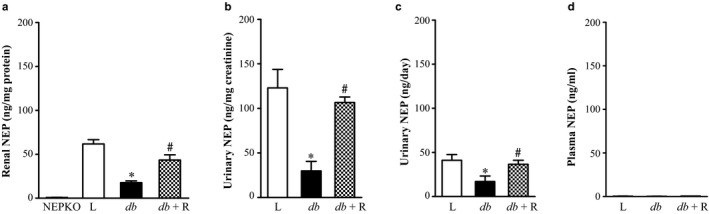
Renal and urinary NEP levels were decreased in *db/db* diabetic mice. ELISA was used to measure (a) renal NEP, (b) urinary NEP/creatinine, (c) daily urinary NEP excretion, and (d) plasma NEP/ml in lean control (L), untreated *db*/*db* diabetic (*db*), and *db*/*db* diabetic mice treated with rosiglitazone (*db+ *R) for 10 weeks. Kidney lysate samples from NEP KO mice were used as negative control in figure a. Values are represented as mean ± *SEM* (*n* = 5–6, *p < .0001 vs. lean controls; ^#^ *p* < .001 vs. untreated *db/db* diabetic mice)

Mouse neprilysin Duoset ELISA kit was used for quantitative determination of renal NEP levels. The assay was validated for specificity by using kidney lysate from NEPKO mice. Kidney lysates were diluted (1:50–1:100) in assay diluent before use. Renal NEP content was significantly decreased in *db/db* mice compared to age matched lean control mice (Figure 4a, *p* < .0001). Treatment with rosiglitazone in *db*/*db* mice normalized the renal NEP levels. Urinary NEP/creatinine ratio was significantly decreased in *db/db* mice compared to age‐matched lean control mice (Figure 4b, *p* < .0001). Rosiglitazone treatment normalized the NEP/creatinine ratio (Figure 4b, *p* < .0001). 24‐hr NEP excretion significantly decreased in untreated *db/db* mice compared to lean control mice (Figure 4c, *p* < .05). Since plasma NEP levels are low, samples were diluted only (1:1). There was no significant difference in plasma NEP levels between lean control, untreated *db*/*db* and rosiglitazone‐treated *db*/*db* diabetic mice (0.4 ± 0.1, 0.38 ± 0.1, 0.5 ± 0.1 ng/ml). As shown in Figure 4d, the values were expressed using the same scale for renal and urinary NEP. The values for the three groups were <0.5 ng/ml, which is at the lower end of the standard curve, but within the limits of the assay sensitivity (0.1–6 ng/ml). It is worth noting that the NEP levels for urine and renal NEP were almost 100‐ folds more than plasma NEP.

### 3.5 Effect of rosiglitazone on renal NEP activity

**Figure 5:**
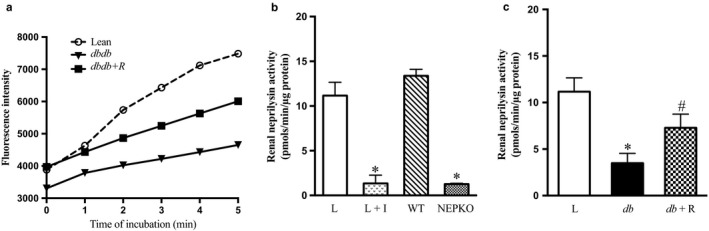
Renal NEP enzymatic activity was decreased in *db/db* diabetic mice. For validation of NEP activity, the assay was performed at different time of incubation using NEP inhibitor (Thiorphan) and kidney lysate from NEP knockout (NEPKO). (a) Time in minutes versus fluorescence intensity in lean control (Lean), untreated *db/db* diabetic (*dbdb*), and *db/db* diabetic mice treated with rosiglitazone (*dbdb*+ R). (b) Renal NEP activity in lean control (L), lean + NEP inhibitor (Thiorphan) (L + I), Wild type (WT), and NEPKO. (c) Renal NEP activity in lean control (L), untreated *db/db* diabetic (*db*), and *db/db* diabetic mice treated with rosiglitazone (*db*+ R) for 10 weeks. Values are represented as mean ± *SEM* (*n* = 6–9, *p < .001 vs. lean controls & WT; ^#^ *p* < .001 vs. untreated *db/db* diabetic mice)

The SensoLyte® 520 Neprilysin Assay Kit was used for ascertaining functional nature of the enzyme in the renal and urinary samples. Samples of kidney lysates (0.5–1 µg of protein) were incubated with 15 µl of assay buffer and NEP fluorogenic substrate for 6 hr. As shown in Figure 5a, there was a time‐dependent increase in NEP activity in all kidney samples. Treatment of kidney lysate with different concentration of rosiglitazone in vitro, had no effect on renal NEP activity (data not shown). We used thiorphan and NEPKO kidney lysates as negative controls to validate the NEP activity assay kit. As expected, incubation with thiorphan inhibited NEP activity in lean nondiabetic controls and there was minimal NEP activity in NEPKO kidney lysates (Figure 5b, *p* < .0001). In agreement with the western blot data, there was a significant decrease in renal NEP activity in *db/db* mice compared with control mice (Figure 5c, *p* < .001). Treatment of *db*/*db* diabetic mice with rosiglitazone for 10 weeks significantly increased NEP activity compared with untreated *db*/*db* diabetic mice (Figure 5c, *p* < .001).

### 3.6 Effect of rosiglitazone on renal NEP immunostaining

**Figure 6:**
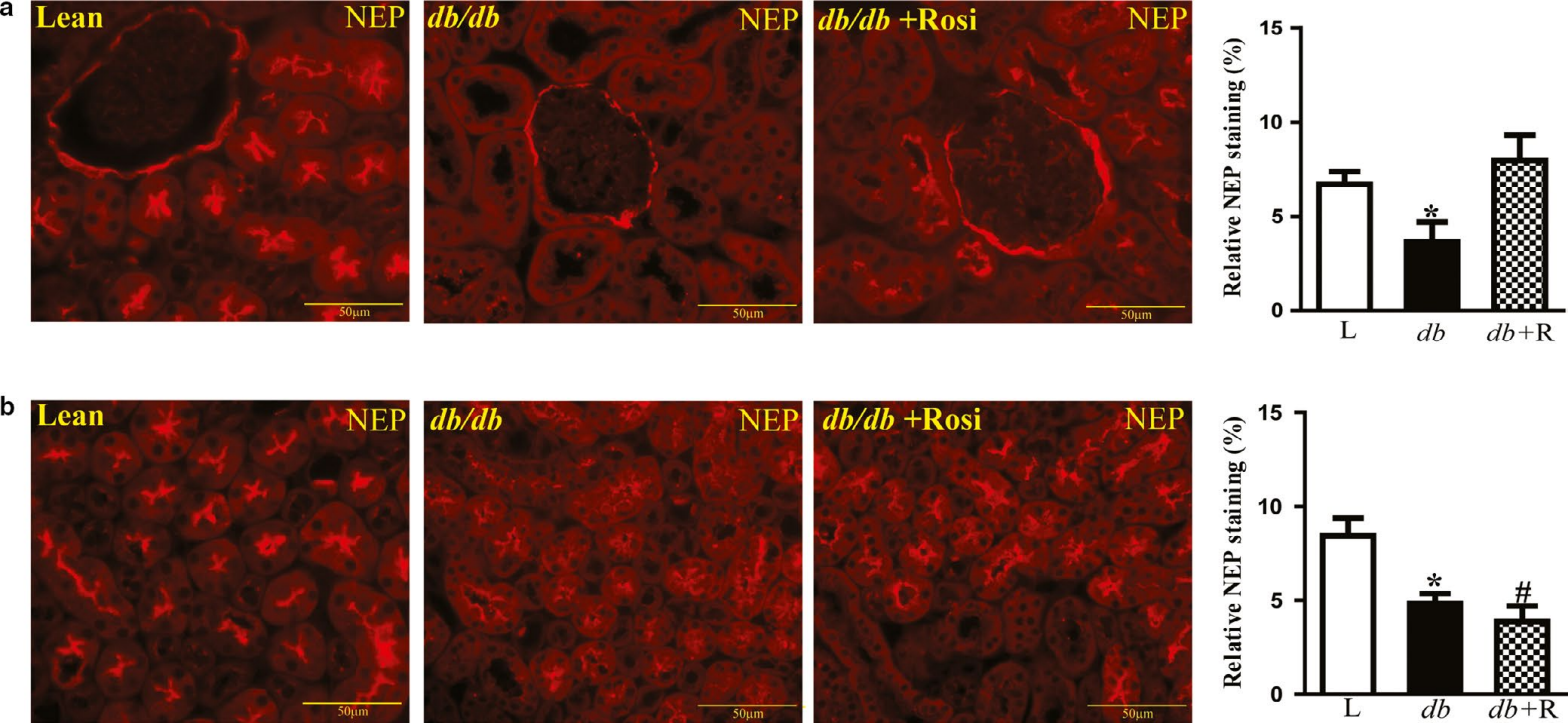
Immunofluorescence staining of renal NEP in the cortex and medulla of lean control, *db*/*db* diabetic, and *db*/*db* diabetic mice treated with rosiglitazone (*db*/*db*+ Rosi) for 10 weeks. Staining shows strong immunoreactivity in the brush border of the proximal tubules, distal tubules, and the Bowman capsules. Relative NEP immunostaining was quantified in cortex and medulla using Metamorphe software. Analysis showed significant decrease in NEP immunostaining in *db*/*db* diabetic mice compared to controls (*p < .001 vs. lean control, ^#^ *p* < .001 vs. lean control). Rosiglitazone treatment normalized NEP expression in the cortex and not in the medulla. Magnification: 40×, scale bars: 50 μm

Kidney sections were stained with corresponding antibodies to examine the effects of type‐2 diabetes and rosiglitazone treatment on renal NEP distribution. In the kidneys of lean control mice, NEP protein expression was observed in the cortex (Figure 6a, red fluorescence) and medulla (Figure 6b) predominantly in the Bowman's capsule and in the brush border of the proximal convoluted tubules, and to a lesser extent in the mesangial. While in *db/db* mice, NEP protein was widely expressed in the Bowman's capsule and to a lesser extent in the brush border of the proximal tubules compared to control mice. In the cortex, there was a significant decrease in NEP expression among the untreated *db/db* mice compared to the treated mice (Figure 6a, *p* < .001). Similar to the cortex, protein expression of NEP in the medulla was significantly reduced in *db/db* compared to control mice (Figure 6b, *p* < .001). However, rosiglitazone has no effect on renal NEP in the medulla.

### 3.7 Renal NEP and ADAM17 immunostaining

**Figure 7:**
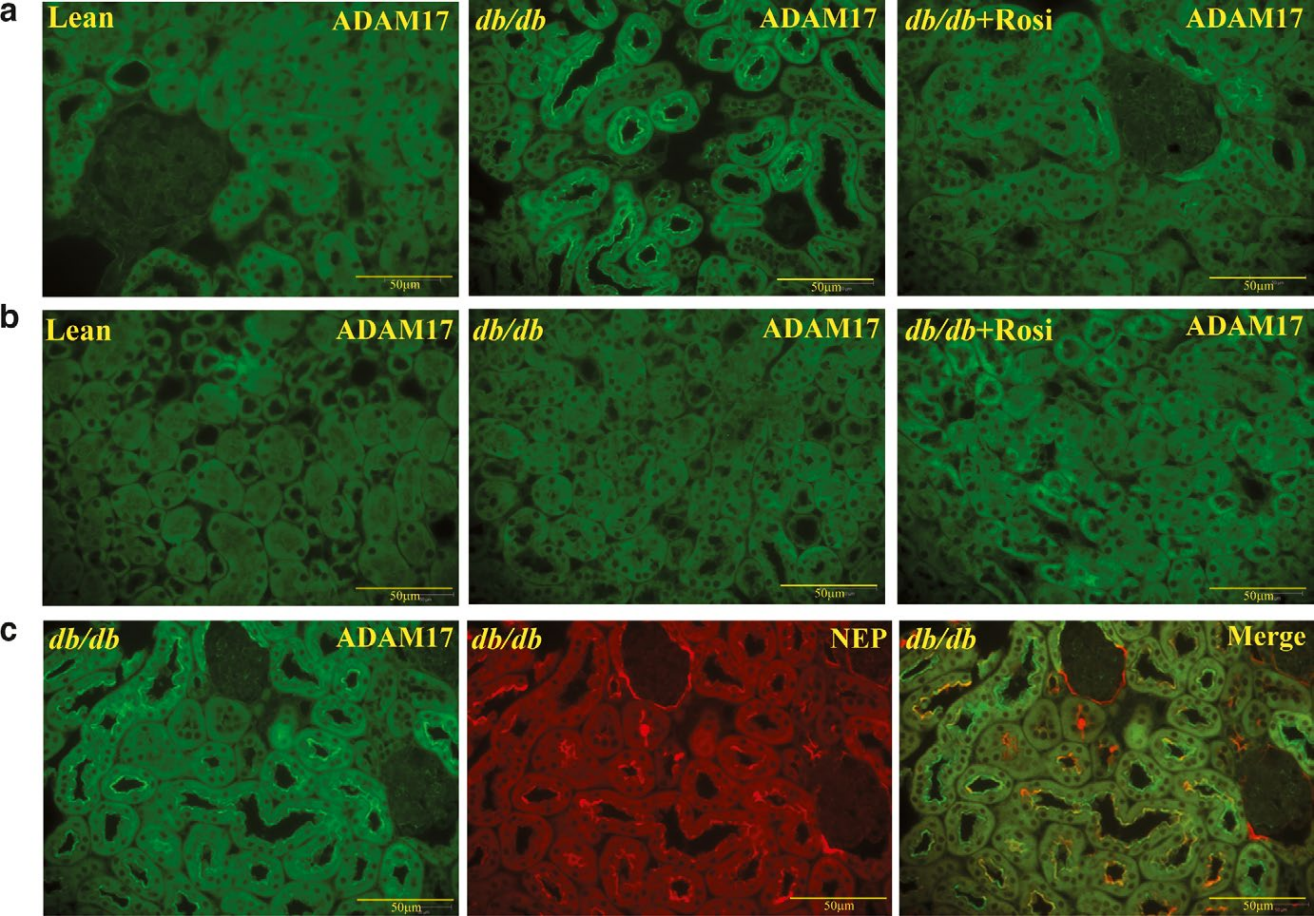
Immunofluorescence staining for renal ADAM 17 shows strong immunoreactivity in the renal tubules of the cortex (a) and medulla (b) of lean control, *db*/*db* diabetic, and *db*/*db* diabetic mice treated with rosiglitazone (*db*/*db*+ Rosi) for 10 weeks. 40× magnification. ADAM17 immunoreactivity was mainly observed in the *db*/*db* diabetic kidney, specifically in the brush border of the proximal and the distal convoluted tubules. (c) Double immunofluorescence staining for ADAM 17 (green) and NEP (red) in cortex of *db*/*db* diabetic mice shows significant overlapping reactivity and co‐localization in the proximal renal tubules (magnification: 40×, scale bars: 50 μm)

ADAM17 protein expression was observed mainly in the brush border of proximal convoluted tubules and distal tubules in *db/db* diabetic mice (Figure 7a, green fluorescence). Treatment with rosiglitazone decreased ADAM17 in *db/db* mice (Figure 6a). ADAM17 expression was not observed in the medulla of *db/db* diabetic mice (Figure 7b). ADAM 17 and NEP were found to be colocalized in the tubular region of the *db/db* mice kidney cortex (Figure 7c, yellow fluorescence).

## 4 DISCUSSION

To the best of our knowledge, this is the first study to investigate the regulation of renal and urinary NEP in *db/db* diabetic mice and to demonstrate their modulation by treatment with the PPARγ agonist, rosiglitazone. Renal NEP protein expression and activity were significantly reduced in 16‐week‐old *db/db* diabetic mice compared to their age‐matched controls. There is a basal level of urinary NEP in the nondiabetic lean control mice, which was significantly decreased in *db*/*db* diabetic mice. These data suggest that urinary NEP could be used as an index for intrarenal NEP status. Treatment with the anti‐diabetic medication rosiglitazone normalized glycemia, albuminuria, and renal NEP protein expression. Chronic treatment of nondiabetic lean mice with rosiglitazone had no effect on blood glucose or renal NEP expression and activity. Therefore, the study was focused on the effect of hyperglycemia and rosiglitazone on NEP in the *db*/*db* mice. Using both western blot analysis and quantitative MALDI‐TOF imaging, we have previously shown decreased renal NEP in streptozotocin‐induced (STZ) diabetic mice (Somineni, Grobe, Chodavarapu, & Morris, 2012). In agreement with our data, a significant reduction of renal NEP activity and expression was also reported in female hypertensive STZ diabetic mRen2.Lewis rat (Yamaleyeva et al., 2012) and DOCA‐ salt hypertensive rats (Bae, Kim, Ma, & Kim, 2009). It is interesting that renal NEP was unchanged in male hypertensive STZ diabetic mRen2.Lewis rats (Yamaleyeva et al., 2012). Furthermore, urinary exosomes isolated from the Zucker diabetic fatty rats showed no difference in NEP protein expression compared to non‐diabetic controls (Raimondo et al., 2013).

In diabetic conditions, overactivation of RAS has been shown to cause several complications, including cardiovascular dysfunctions (Brewster et al., 2003) and DKD (Roscioni, Heerspink, & Zeeuw, 2014). NEP has been found to play a pivotal role in the degradation of a range of physiologically important peptides such as Ang I (Rice et al., 2004), β amyloid (Guan, Chow, Shah, Rhodes, & Hersh, 2012), bradykinin, and natriuretic peptides (Judge et al., 2015). The accumulation of islet‐amyloid polypeptide in pancreatic cells and β amyloid in neuronal cells lead to cellular apoptosis characteristics of type 2 diabetes and Alzheimer's disease, respectively (Guan et al., 2012). A previous study has shown that amyloid and tau pathologies associated with Alzheimer's disease are significantly reduced after chronic treatment with rosiglitazone (Escribano et al., 2010). Recently, we have shown treatment with the NEP inhibitor LBQ657 reduced Ang‐(1–7) in vivo and identified NEP as a major source of renal Ang‐(1–7) in mice and humans (Domenig et al., 2016). These studies, in support with our results lead us to suggest that downregulation of renal NEP in *db/db* mice may contribute to the pathogenesis of diabetic kidney disease. Interestingly, treatment with estrogen in hypertensive transgenic Ren2 rat model upregulated NEP expression and activity in the uterus with no observed effect in the kidneys, indicating that NEP activity regulation is tissue specific (Neves et al., 2006).

In recent years, new therapeutic strategies to improve outcomes for patients with heart failure involving NEP inhibition were studied. Combined ACE/NEP inhibitor (omapatrilat) versus ACE inhibitor (quinapril) in diabetic apolipoprotein E‐knockout mice showed that omapatrilat had greater blood pressure‐lowering and renoprotective effect (Jandeleit‐Dahm et al., 2005). However, omapatrilat was associated with high incidence of fatal angioedema and therefore was withdrawn (Judge et al., 2015). The combination of NEP inhibitor with either ACE or endothelial converting enzyme (ECE) inhibitors showed a reduction of blood pressure and albuminuria in streptozotocin‐induced diabetic Sprague‐Dawley rats, but NEP inhibition alone failed to have a similar effect (Tikkanen et al., 2002). TG(mRen2)27 (Ren2) transgenic rats overexpress the mouse renin gene, manifested hypertension and increased tissue Ang II level (Whaley‐Connell et al., 2006). Treatment of Ren2 with the angiotensin‐type 1 receptor (AT1R) blocker, valsartan has been shown to reduce albuminuria, high blood pressure and increase renal ACE2 and NEP mRNAs expression (Whaley‐Connell et al., 2006). These data suggest that ACE2 and NEP could contribute to the renoprotective effects of valsartan by increasing the formation of Ang‐(1–7) and opposing the effect of Ang II (Whaley‐Connell et al., 2006).

Our previous studies have shown increased shedding of enzymatically active renal ACE2 and NEP in the urine of type 2 diabetic mice (Chodavarapu et al., 2013; Somineni et al., 2014) and in type 2 diabetic patients (Gutta et al., 2018). We have also shown colocalization of ACE2 and ADAM 17 in the brush border of renal proximal tubules in Akita and *db*/*db* diabetic mice (Chodavarapu et al., 2013; Salem, Grobe, & Elased, 2014). Recent studies suggest a role of ADAM17 in the shedding of soluble, active NEP into the media of endothelial cells (Kuruppu, Rajapakse, Minond, & Smith, 2014). In this study, we have shown for the first‐time colocalization of renal NEP and ADAM 17 immunostaining in the brush border of the proximal tubules. Although the exact source of urinary NEP is not currently known, NEP may be actively shed for renal tubules by ADAM 17.

Recently, the combined treatment with an angiotensin receptor blockade and NEP inhibition is a potential new therapy for patients with heart failure and reduced ejection fraction (McMurray et al., 2014). LCZ696, an angiotensin (AT1) receptor blocker‐neprilysin inhibitor (ARNi), in clinical studies showed a higher efficacy in hypertension management (Gu et al., 2010; Minguet, Sutton, Ferrero, Gomez, & Bramlage, 2015). The PARADIGM‐HF trial showed that treatment with LCZ696 in heart failure patients significantly reduced the rate of deaths and hospitalization from cardiovascular causes compared to ACE inhibitor (McMurray et al., 2014). However, the PARAMOUNT study (Prospective Comparison of ARNI [angiotensin receptor‐neprilysin inhibitor] with ARB on Management of Heart Failure with Preserved Ejection Fraction) trial reported that this combination impacts renal function as shown by an increase in urinary albumin to creatinine ratio (UACR)‐mediated through the effects of increased bioavailability of natriuretic peptides (NPs) by NEP inhibition (Voors et al., 2015). Furthermore, increased levels of atrial natriuretic peptide (ANP) have been shown to contribute to glomerular hyperfiltration, suggesting potential renal dysfunction in LCZ696‐treated patients (Ortola et al., 1987). Although detailed and long‐term effects of NEP inhibition need to be studied, the studies above support our conclusion that NEP might have a role in kidney function of diabetic mice (Damman et al., 2018; Ruggenenti & Remuzzi, 2015). Thus, new perspective treatment of heart failure with LCZ696 might oppose the protective effect of NEP in other organs such as brain, pancreatic beta islet cells, and kidneys. Further studies for NEP inhibitory effects need to be addressed in some diseased populations such as in patients with Alzheimer's and diabetes.

Randomized controlled trials suggest that strict glycemic control can reduce the progression of DKD (Turner, 1998). In this study, we investigated the effects of glycemic control with rosiglitazone on albuminuria and NEP expression in *db/db* mice. In the same diabetic model, our previous studies demonstrated a positive correlation between increased shedding of renal ACE2 into urine and albuminuria, reflecting the progression of diabetic kidney injury (Chodavarapu et al., 2013). Treatment of *db*/*db* diabetic mice with rosiglitazone attenuated renal injury and decreased urinary ACE2 and renal ADAM17 protein expression (Chodavarapu et al., 2013). Furthermore, insulin treatment of Akita diabetic mice normalized hyperglycemia and attenuated urinary ACE2 shedding, albuminuria, and renal ADAM17 expression (Salem et al., 2014). In contrast with this study, rosiglitazone normalized renal and urinary NEP expression in *db/db* mice, while the reduced renal NEP activity in *db*/*db* mice was altered by rosiglitazone treatment.

Despite the protective effect of rosiglitazone in DKD reported in several studies (Jia et al., 2014), rosiglitazone usage was restricted because of its suspect association with heart failure (Nissen & Wolski, 2007). This study showed that rosiglitazone upregulates NEP, but it is not clear if NEP was also increased in the heart, which has not been investigated in this study. If there was evidence that NEP increased in heart failure, it can be speculated that incidence of heart failure and myocardial infarction associated with rosiglitazone could be mediated through the upregulation of NEP expression. However, it has recently been shown that patients with heart failure with preserved ejection fraction have lower circulation of NEP levels than control individuals with no evidence of diastolic dysfunction or heart failure (Lyle et al., 2020). This data questions the assumption that soluble NEP is a potential biomarker for heart failure patients with reduced ejection fractions, contrary to its recent importance in heart failure with reduced ejection fraction (Lyle et al., 2020). In the present study, there was no significant difference between lean controls and diabetic mice. In addition, plasma NEP levels were almost 100 times lower than renal and urinary NEP.

The current indicators in the evaluation of DKD are measurements of urinary albumin excretion (UAE) and estimated GFR (eGFR) (Jha, Jandeleit‐Dahm, & Cooper, 2014). However, studies have reported limitations in using albuminuria as an early and sensitive biomarker (Lee & Choi, 2015). We and others suggested that urinary ACE2 and NEP could be a potential biomarker for CKD.

In conclusion, data support the hypothesis that hyperglycemia decreased intrarenal NEP expression and activity, which could have a pivotal role in the pathogenesis and progression of DKD. Urinary NEP could be used as an index of intrarenal NEP status. Data also suggest that the renoprotective effects of rosiglitazone in the *db*/*db* mice could be mediated by upregulation of renal NEP expression and activity. It is tempting to speculate that changes in urinary NEP levels could be used as a biomarker for the development and progression of DKD.
